# Accuracy of a continuous glucose monitoring system applied before, during, and after an intense leg-squat session with low- and high-carbohydrate availability in young adults without diabetes

**DOI:** 10.1007/s00421-024-05557-5

**Published:** 2024-07-22

**Authors:** Manuel Matzka, Niels Ørtenblad, Mascha Lenk, Billy Sperlich

**Affiliations:** 1 Integrative and Experimental Exercise Science & Training, Institute of Sport Science, University of Würzburg, Würzburg, Germany; 2 Department of Sports Science and Clinical Biomechanics, University of Southern Denmark, Odense, Denmark

**Keywords:** Nutrition, Technology, Strength training, Metabolism

## Abstract

**Purpose:**

The aim was to assess the accuracy of a continuous blood glucose monitoring (CGM) device (Abbott FreeStyle Libre 3) against capillary blood glucose measurement (BGM) before, during, and after an intense lower body strength training session in connection with high- versus low-carbohydrate breakfasts.

**Methods:**

Nine adults (22 ± 2 years) completed a strength training session (10 × 10 at 60% 1RM) twice after high-carbohydrate and twice after low-carbohydrate breakfasts. CGM accuracy versus BGM was assessed across four phases: post-breakfast, pre-exercise, exercise, and post-exercise.

**Results:**

Overall fed state mean BGM levels were 84.4 ± 20.6 mg/dL. Group-level Bland–Altman analysis showed acceptable agreement between CGM and BGM across all phases, with mean biases between − 7.95 and − 17.83 mg/dL; the largest discrepancy was in the post-exercise phase. Mean absolute relative difference was significantly higher post-exercise compared to pre-exercise and exercise phases, for overall data and after the high-carbohydrate breakfast (all *p* ≤ 0.02). Clark Error Grid analysis showed 50.5–64.3% in Zone A and 31.7–44.6% in Zone B, with an increase in treatment errors during and after exercise.

**Conclusion:**

In this group of healthy participants undergoing strength training, CGM showed satisfactory accuracy in glucose monitoring but varied substantially between individuals compared to BGM and fails in meeting clinical criteria for diabetic monitoring. CGM could aid non-diabetic athletes by tracking glucose fluctuations due to diet and exercise. Although utilization of CGM shows potential in gathering, analyzing, and interpreting interstitial glucose for improving performance, the application in sports nutrition is not yet validated, and challenges in data interpretation could limit its adoption.

**Supplementary Information:**

The online version contains supplementary material available at 10.1007/s00421-024-05557-5.

## Introduction

Continuous interstitial glucose monitoring (CGM) represents a significant technological advancement in diabetes management, offering numerous benefits over traditional blood glucose sampling methods. Primarily, CGM enables the assessment of blood glucose levels with a typical delay of 10–20 min (Kovatchev et al. 2009) corresponding to the time needed for glucose to manifest in the interstitial fluid, levels of which have been shown to closely correlate with blood glucose concentrations (Holzer et al. 2022). The prompt monitoring enables individuals with diabetes to make immediate adjustments to their diet, physical activity, or medication protocol (Vigersky and Shrivastav 2017). With this continuous feedback loop, better glycemic control can be achieved, reducing the risk of both hyperglycemia and especially hypoglycemia (Moser et al. 2020). Additionally, the data collected over time from CGM devices can provide valuable insights into the effects of lifestyle choices on blood glucose levels, assisting in more personalized and effective diabetes management (Vigersky and Shrivastav 2017).

Although CGMs were initially designed to assist in the clinical management of diabetes, there is now emerging interest in the application of real-time glucose monitoring to athletic populations to optimize performance, nutrition, and recovery (Bauhaus et al. 2023; Bowler et al. 2023; Flockhart and Larsen 2024; Holzer et al. 2022; Klonoff et al. 2023). Within this, companies that distribute CGM along with smartphone applications claim that the CGM can provide information on the success of various nutrition strategies and optimize performance and advertised with statements such as 'Master your metabolism', 'Unlock the power of glucose' (TT1 PRODUCTS 2024), and 'Fuel better for your next workout' (Ultrahuman Healthcare 2024). Potentially, CGM may assist athletes to understand how their bodies respond to different types of exercises, training intensities, and dietary choices. However, it is crucial to emphasize that these claims regarding the potential utility of CGM in sports for non-diabetic individuals are not substantiated by evidence-based justification and the potential utilization of CGMs in tailoring nutritional strategies remain unverified and challenges in data accuracy and interpretation may hinder athletes' adoption of CGMs. More investigation is needed to grasp the reliability of data derived from CGM before we can assert that the information holds significance for athletes who may be prone to falling for marketing exaggerations or simplified explanations.

Given the use in both clinical and athletic settings, assessing the accuracy of CGM becomes paramount, especially in relation to strength training and dietary variations. Strength training is a fundamental training routine in many sports and can significantly impact glucose metabolism and insulin sensitivity, which may influence the accuracy and responsiveness of glucose sensors (Bolla and Priefer 2020; Lodwig et al. 2003). For instance, a study comparing blood glucose responses between strength and endurance training has observed a smaller initial decrease in blood glucose levels during the activity but more sustained reductions in post-exercise glycemia in strength training (Yardley et al. 2013). However, this study recruited participants having type-1 diabetes, whose glucose metabolism markedly differs from those of individuals without diabetes. To date, the accuracy of CGM during strength training has not been analyzed in healthy individuals without diabetes. Similarly, the consumption of meals with varying macronutrient compositions, such as high-carbohydrate or high-protein intake, which are quite common in athletes engaging in strength training, can lead to distinct glycemic responses. Therefore, evaluating the accuracy of CGM devices under these varying conditions may be of interest and is essential to ensure a high-level of confidence for possible decision-making. Although manufacturers already overconfidently emphasize the overall value of CGM, to the best of our knowledge, no study so far has comprehensively assessed the accuracy of CGM devices in the context of strength training and in connection with the consumption of meals with diverse macronutrient profiles in individuals without diabetes.

Therefore, the aim of this study was to investigate the accuracy of a CGM device compared to capillary blood glucose measurement (BGM) for monitoring blood glucose levels before, during and after an intense lower body strength training and the influence of a high- compared to a low-carbohydrate breakfast in young adults without diabetes.

## Materials and methods

### Experimental overview

**Fig. 1:**
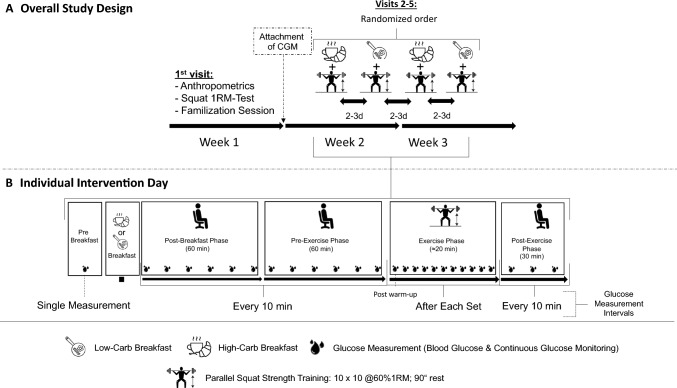
Schematic representation of **A** the overall study design and **B** the procedure during an individual intervention day

The overall study design is illustrated in Fig. 1. All participants visited the laboratory five times over a 3-wk period. In the first week, each participant underwent a one-repetition maximum (1RM) test in the smith-machine parallel squat and anthropometric data (Table 1) were collected.

**Table 1:** Study participants’ characteristics

	Age [years]	Body mass [kg]	Body height [cm]	Body mass index [kg/m^2^]	Muscle mass [kg]	Body fat [%]	Squat 1RM [kg]
All (*n* = 9)	22.2 ± 1.9	70.4 ± 10	171 ± 10	24.0 ± 2.0	51.9 ± 9.7	21.1 ± 8.5	79.3 ± 28.2
Females (*n* = 5)	21.8 ± 1.3	65.0 ± 9.9	165 ± 8	23.8 ± 2.0	44.5 ± 3.9	27.2 ± 5.3	58.0 ± 11.2
Males (*n* = 4)	22.8 ± 2.3	77.2 ± 4.9	180 ± 6	24.2 ± 2.0	61.2 ± 6.2	13.6 ± 4.8	105.9 ± 19.1

Values expressed as mean ± standard deviation

*1RM* one-repetition maximum

In week 2 and 3, all visited the laboratory twice to perform a strength training following a high-carbohydrate breakfast and twice to perform a strength training (i.e., Session 1 and 2) following a low-carbohydrate breakfast. The breakfast condition was randomly allocated. The visits were separated by at least 2–3 days. Each participant attached the CGM patch according to the manufacturer’s guidelines the day before the first laboratory visit. The participants arrived in the morning in a fasted state (i.e., no breakfast, with only water permitted). Each intervention visit followed the same protocol as implemented before (Clavel et al. 2022), which is briefly outlined as follows: after arrival, BGM and CGM values were measured before breakfast in a fasted state. The subjects then consumed, in randomized order, either the standardized high-carbohydrate or low-carbohydrate breakfast. Immediately after, the regular recording of both the BGM and the CGM began. For the first 120 min after breakfast, the participants remained seated with BGM and CGM recording every 10 min. Immediately afterward, the standardized strength training began, with BGM and CGM recordings immediately after the warm-up and after each working set. In the 30 min after the strength training, BGM and CGM were recorded every 10 min. The morning with timing of breakfast and the strength training routine was split into four distinct phases for statistical analysis as follows and based on the enclosed rationales: (i) The ‘post-breakfast’ period, defined as the first 60 min following breakfast consumption, was selected due to anticipated significant fluctuations in blood glucose levels among young, healthy adults, with peak levels typically occurring approximately 30–45 min after breakfast (Clavel et al. 2022; Hostmark et al. 2006); (ii) the ‘pre-exercise’ phase, defined as the 60 min preceding the strength training session, was selected based on literature indicating that rates of change in blood glucose levels are expected to revert close to pre-breakfast conditions in young, healthy adults (Clavel et al. 2022; Hostmark et al. 2006); (iii) the 'exercise' phase, encompassing both the warm-up and the lower body strength training, was designated to analyze the distinct blood glucose responses observed during the strength training sessions (Yardley et al. 2013); (iv) the 'post-exercise' phase, beginning immediately after the final set of the strength training and extending for 30 min, was analyzed due to the expected distinct blood glucose fluctuations immediately following the strength training session (Yardley et al. 2013). During post-breakfast, pre-exercise, and post-exercise, all participants remained seated.

### Subjects

Nine (5 females) young recreationally active physical education students without diabetes were recruited for the study, who volunteered to participate without receiving any compensation. All participants were required to have prior familiarity with the squat exercise. Before the study, five participants regularly engaged in lower body strength training in a gym (1–2 times per week), while the other four participants were only engaged in sports/physical activities involving the lower limbs (i.e., acrobatics, running). The main characteristics of the study group are summarized in Table 1.

During the period of investigation, all participants were asked to refrain from alcohol and to maintain their nutritional habits the evening before each testing day and to ensure a minimum of 8 h of sleep. The study participants were informed in advance about the study objective and possible risks, and informed consent was obtained from the participants before the start of the study. The study was approved by the faculty’s ethics committee (EV2024/1-3004) and conducted in concordance with the Declaration of Helsinki.

### Procedures

#### Capillary blood glucose measurement (BGM)

For capillary blood glucose measurements, 20 μl capillary blood was sampled at each of the previously described time intervals from the right ear lobe and was analyzed using the EKF Biosen C-Line, (EKF Diagnostics Holdings plc, Penarth, UK), which has shown high agreement (3.5% ± 3.4%) with a gold standard reference without any concentration-dependent changes (slope of regression line: 0.03 ± 0.06 to 0.09 ± 0.06) (Nowotny et al. 2012).

#### Continuous glucose monitoring

For CGM, all participants used a FreeStyle Libre 3 CGM sensor (Abbott, Chicago, USA). Throughout the intervention period, CGM recorded interstitial glucose values concurrently with each instance of BGM measurement. All CGM data were stored by the accompanying app (“Libre 3” by Abbott) before analysis of data. Each participant attached the CGM patch and installed the app for data recording according to manufacturer guidelines the day before the first laboratory visit. The patch was attached at the back of the upper arm on the side of the participant's choice. When attaching a CGM sensor, a hair-thin needle is inserted under the skin which measures glucose levels in the interstitial fluid.

### Anthropometric measurements

Anthropometric data of the participants were collected during the first laboratory visit in week one. Each participant’s body mass, muscle mass and body fat percentage were obtained with an eight-point bioimpedance scale (Tanita BC-601, Tanita Europe B.V., Amsterdam, The Netherlands). Body height was assessed using a stadiometer (seca GmbH and Co. KG, Hamburg, Germany).

#### Macronutrient content of breakfast

Based on the methodology outlined previously regarding CGM validity (Clavel et al. 2022), the two distinct standardized meals in this study were composed and portioned relative to each participant's body mass. The high-carbohydrate meal (High-Carb) contained 1 g of carbohydrates per kg body mass ceiling at 70 g per meal containing of toast, jam, and orange juice. The low-carbohydrate (Low-Carb) meal was isocaloric compared to high-carb breakfast and consisted of cottage cheese with nuts. For example, for a person with a body mass of 60 kg the high-carb breakfast contained 60 g, 6 g, 2 g (= 296 kcal) and low-carb 5 g, 25 g, 19 g (= 300 kcal) for carbohydrates, protein, and fat, respectively.

#### Strength training session

In the first week, all participants underwent a 1RM test in the smith-machine parallel squat (SCHNELL Trainingsgeräte GmbH, Gachenbach, Germany) in accordance with the protocol described by Haff and Triplett (2015). After a 30-min rest period after the 1-RM test, a resistance training session was conducted for familiarization. This session entailed 5 sets of 10 repetitions at 60% of 1RM, interspersed with 90-s rest intervals. The lifting mass was adjusted based on the participant's ability at the end of the fifth set. If participants could perform more than 4–5 repetitions beyond the fifth set, the lifting mass was increased by 5–10%. Conversely, if muscular failure was experienced within these sets, the mass was reduced by a similar margin.

During the intervention in week 2 and 3, all participants initiated their lower body strength training session with a warm-up following the pre-exercise phase. The warm-up routine included 2 sets of 20 bodyweight squats and 1 set of 5 parallel squats in the smith machine at 50% of their 1RM, with 60-s rest between each warm-up set. The main training period involved 10 sets of 10 squats at 60% of the 1RM established in the first week, interspersed with 90-s rest intervals. The prescribed movement speed was 2–3 s for the eccentric (lowering) phase and 1 s for the concentric (lifting) phase, including a 1-s static hold at the bottom reversal point. This reversal point, where the thighs are parallel to the ground, was standardized using a bench that participants had to touch with each repetition. Participants also reported their perceived number of additional possible repetitions using the “repetitions in reserve” method (Remmert et al. 2023) and rated their subjective exertion on the Borg 6–20 scale (Borg 1970). In cases when participants were unable to complete the prescribed 10 repetitions within a set, the lifting mass was adjusted for the subsequent set to ensure completion of the targeted repetitions. Table 2 summarizes the achieved strength training and subjective variables.

**Table 2:** Strength training and subjective parameters for the training sessions with different carbohydrate availability

Training session with	Total repetitions [n]	Volume load [kg]	Repetitions in reserve [*n*]	6–20 Borg Scale [a.u.]
High-carbohydrate breakfast	98.6 ± 12.6	4911 ± 670	3.4 ± 0.5	14.2 ± 1.8
Low-carbohydrate breakfast	99.3 ± 12.7	5009 ± 679	3.6 ± 0.5	13.8 ± 1.8

Values are expressed as mean ± standard deviation

### Statistical analyses

Data were processed and analyzed using R (R-Core-Team 2021). The level of significance was set to *p* < 0.05 for all analyses.

The agreement between CGM and BGM was analyzed using the Bland–Altman analysis, complemented by calculating the limits of agreement (LoA) and associated 95% confidence intervals (CI) (Bland and Altman 1986). The analysis was performed for each of the specific phases, i.e., ‘post-breakfast’, ‘pre-exercise’, ‘exercise’, ‘post-exercise’. The standardized bias was calculated to rate the magnitude of the bias according to the following thresholds: > 0.2 (small), > 0.6 (moderate), > 1.2 (large), > 2 (very large) (Hopkins 2000).

Mean absolute relative difference (MARD) across the phases (‘post-breakfast’, ‘pre-exercise’, ‘exercise’, ‘post-exercise’) and conditions (low and high-carb) was analyzed using non-parametric methods due to non-normal distribution (Shapiro–Wilk test) (Reiterer et al. 2017). The Kruskal–Wallis test assessed differences in MARD across phases overall and within each dietary condition (Kruskal and Wallis 1952). Following this procedure, pairwise comparisons were performed using Wilcoxon rank-sum test to identify significant phase pair differences in each condition (Dunn 1964). In addition, effect sizes were computed using the rank–biserial correlation (*r*) with values of 0.00–0.09, 0.10–0.29, 0.30–0.49, and ≥ 0.50 meaning a trivial, small, medium, and large effect, respectively (Kerby 2014).

Clark Error Grid Analysis (EGA; Clarke et al. 2008) was conducted to evaluate the clinical accuracy of the CGM measures compared to BGM. EGA aims to inform whether or not a CGM value will lead to the same treatment decision as concurrent BGM gold standard reference method (Clarke et al. 2008). Here, paired observations of CGM and BGM are plotted and categorized into zones ranging from A (clinically accurate) to E (potentially dangerous errors), with the focus on zones A and B, indicating clinically acceptable discrepancies. This analysis was performed using the “ega” package in R.

As an additional evaluation of the accuracy of the CGM device, we adhered to the standards set forth by the International Organization for Standardization (ISO) standard 15,197:2013 (harmonized version in the European Union: EN ISO 15197:2015) (Breitenbeck and Brown 2017). These guidelines specify that for a blood–glucose monitoring system for self-testing to be considered accurate, 95% of its results must be within ± 15 mg/dL of the reference method's average measured values for blood glucose concentrations below 100 mg/dL, and within ± 15% for blood glucose concentrations of 100 mg/dL or higher.

## Results

897 pairwise comparisons of the nine subjects were included in the analysis across the four investigated phases (post-breakfast: *n* = 203; pre-exercise: *n* = 199; exercise: *n* = 360; post-exercise: *n* = 101). Overall mean BGM values for all conditions and sessions, as well as overall and session specific mean BGM values for low-carbohydrate and high-carbohydrate condition are highlighted in Table 3. Fasting blood glucose levels before breakfast were 81.5 ± 7.3 mg/dL. For detailed information, the individual mean BGM values for each condition and session are provided as supplementary material (SM1).

**Table 3:** Descriptive statistics of blood glucose measurements during each phase shown for all condition and sessions, overall, for each breakfast condition and for each trial per breakfast condition

Phase	All conditions & sessions [mg/dL]	Low-carbohydrate condition			High-carbohydrate condition		
		Overall [mg/dL]	Session 1 [mg/dL]	Sessions 2 [mg/dL]	Overall [mg/dL]	Session 1 [mg/dL]	Session 2 [mg/dL]
Post-breakfast	96.9 ± 22.2	81.8 ± 8.5	81.3 ± 8.5 [59.6: 98.9]	82.2 ± 8.6 [60.1: 97.3]	111.9 ± 21.4	110.3 ± 20.6 [66.7: 153.7]	113.4 ± 22.2 [75: 167.8]
Pre-exercise	84.5 ± 10.5	80.4 ± 7.1	80.2 ± 8.1 [64.7: 93.6]	80.7 ± 6.2 [66.5: 93.3]	88.6 ± 11.7	90.4 ± 10.9 [72.9: 117.3]	87.1 ± 12.2 [58.2: 127.6]
Exercise	84.9 ± 12.6	85.1 ± 11.2	87.4 ± 13.7 [60.1: 126.8]	83.2 ± 8.2 [63.2: 111]	84.7 ± 13.8	87.4 ± 14.6 [61.3: 139.9]	82.4 ± 12.7 [46.6: 122.5]
Post-exercise	86.6 ± 14.6	87.8 ± 14.2	90.3 ± 18.6 [69.8: 126.5]	85.5 ± 8.1 [70.3: 107.1]	85.5 ± 14.9	85.2 ± 17 [51.9: 117.4]	85.8 ± 13.2 [60.6: 118.3]

All values are expressed as mean ± standard deviation; values for the individual sessions in both breakfast conditions are additionally highlighted with minimal to maximal value range [min: max]

### Bland–Altman analysis

**Fig. 2:**
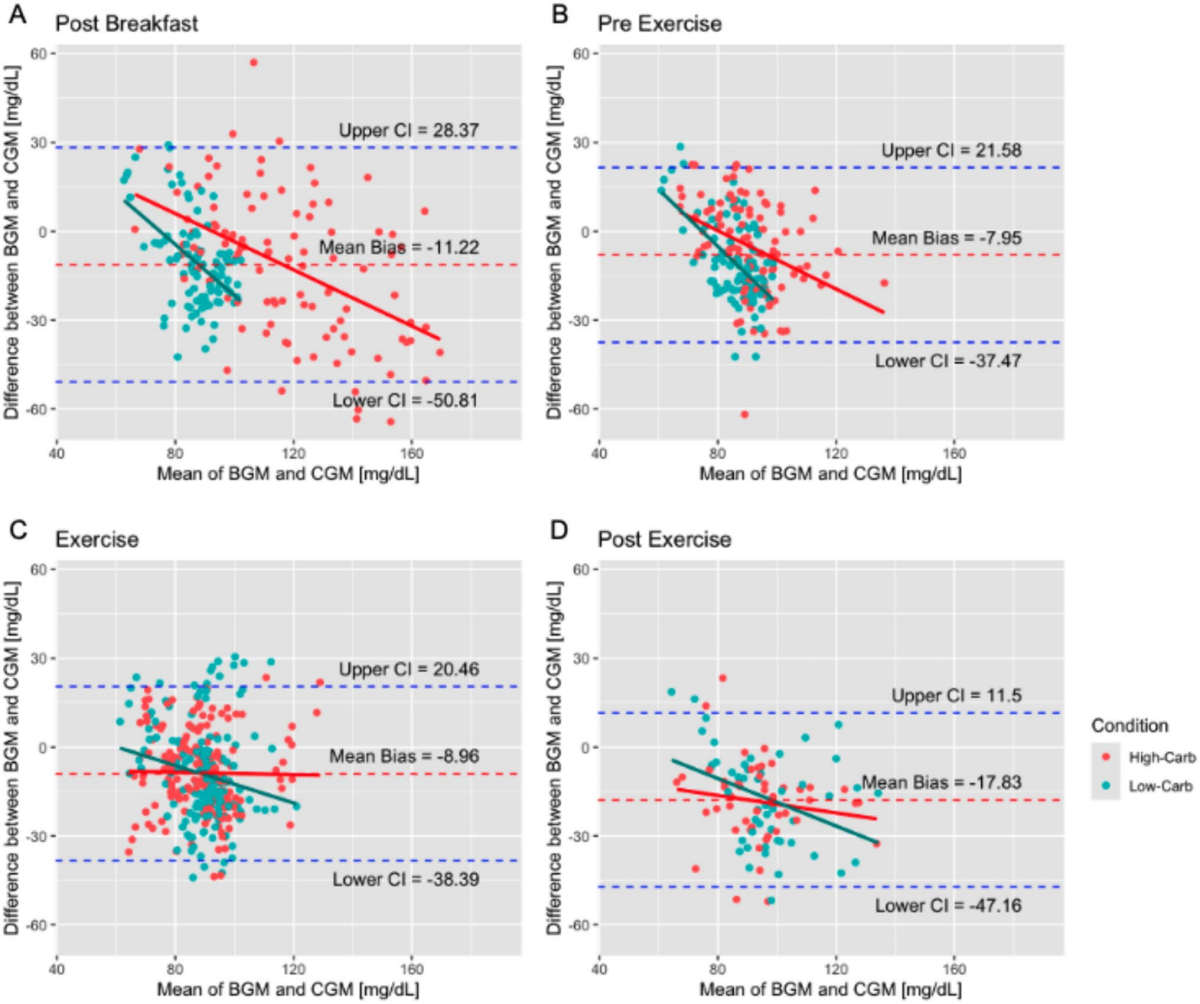
Bland–Altman plots displaying analysis of BGM vs. CGM during the different phases: **A** post-breakfast; **B** pre-exercise, **C** exercise, **D** post-exercise for all training sessions

The Bland–Altman analysis for the different phases is reported as mean bias (CI) and illustrated in Fig. 2. Overall mean biases were small for post-breakfast (− 11.22 [− 14.00 to − 8.44] mg/dL), pre-exercise (− 7.95 [− 10.04 to − 5.85] mg/dL), exercise (− 8.96 [− 10.51 to − 7.41] mg/dL), and moderate for post-exercise (− 17.83 [− 20.75 to − 14.91] mg/dL). Regarding the High-Carb condition, mean biases were small for post-breakfast (− 12.00 [− 16.64 to − 7.35] mg/dL), pre-exercise (− 5.33 [− 8.43 to − 2.23] mg/dL), moderate during exercise (− 8.65 [− 10.67 to − 6.62] mg/dL), and large for post-exercise (− 18.48 [− 22.21 to − 14.76] mg/dL). For Low-Carb, small mean biases were observed for exercise (− 9.28 [− 11.63 to − 6.93] mg/dL) and moderate for post-breakfast (− 10.44 [− 13.48 to − 7.39] mg/dL), pre-exercise (− 10.59 [− 13.32 to − 7.86] mg/dL), and post-exercise (− 17.16 [− 21.700 to − 12.624] mg/dL).

### Mean absolute relative difference

Kruskal–Wallis test showed significant differences in MARD overall (*p* = 0.01) and for high-carb (*p* < 0.01), across different phases, but not low-carb (*p* = 0.61; Table 4). Pairwise comparisons identified significant differences specifically in the ‘exercise’ vs. ‘post-exercise’ and ‘post-exercise’ vs. ‘pre-exercise’ phase both, for overall data (*p* = 0.02 and *p* = 0.01) and for the high-carb condition (*p* = 0.01 and *p* < 0.01).

**Table 4:** Mean absolute relative difference between the continuous glucose monitoring device (CGM) and capillary blood glucose measures (BGM)

Condition	Post-breakfast	Pre-exercise	Exercise	Post-exercise
Overall [%]	19.9 ± 14.2	17.5 ± 13.2*	18.1 ± 13.3*	24.3 ± 17.4
High-carbohydrate breakfast [%]	19.1 ± 15.1	15.7 ± 14.1*	17.1 ± 13.6*	25.0 ± 17.8
Low-carbohydrate breakfast [%]	20.8 ± 13.3	19.3 ± 12.1	19.2 ± 12.9	23.5 ± 17.1

Values are presented as mean ± SD and expressed in percentage differences

*Significantly different from post-exercise (*p* ≤ 0.02)

#### Error grid analysis (EGA)

**Fig. 3:**
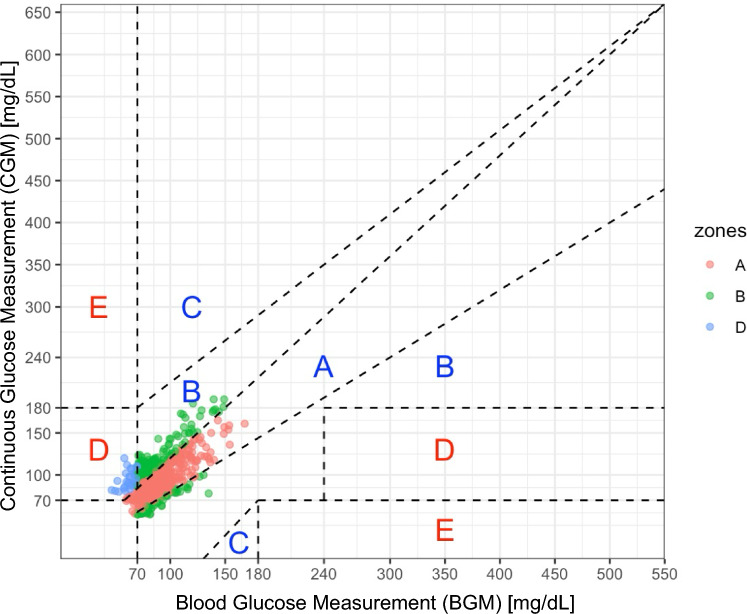
Error grid analysis (EGA) for all phases and conditions (Zone A = clinically accurate; Zone B = clinically acceptable; Zone C = overcorrection/undercorrection; Zone D = potentially dangerous; Zone E = erroneous)

Figure 3 highlights the results of the EGA. Across both conditions, accurate (Zone A) and benign errors (Zone B) encompassed the majority of readings post-breakfast (97.5%), pre-exercise (97.5%), and post-exercise (95%). During exercise, 93.9% of the readings remained in these optimal zones. However, there was an increase in failure to treat errors (Zone D) during exercise (6.1%). During high-carb, post-breakfast (100%) and pre-exercise (99%) readings were mostly accurate or with benign errors. During the exercise phase, accuracy slightly declined with 93.3% in Zone A and B, with Zone D errors increasing to 6.7%. Post-exercise, the accuracy further declined to 92.2% in Zone A and B, with a notable increase in Zone D errors (7.8%). In contrast, during low-carb EGA showed a more stable pattern during exercise (5.6% Zone D) compared to post-breakfast and pre-exercise (5.0% and 4.0% in Zone D, respectively) and lowest Zone D proportions during post-exercise (2.0%).

### ISO 15197:2013/EN ISO 15197:2015

**Fig. 4:**
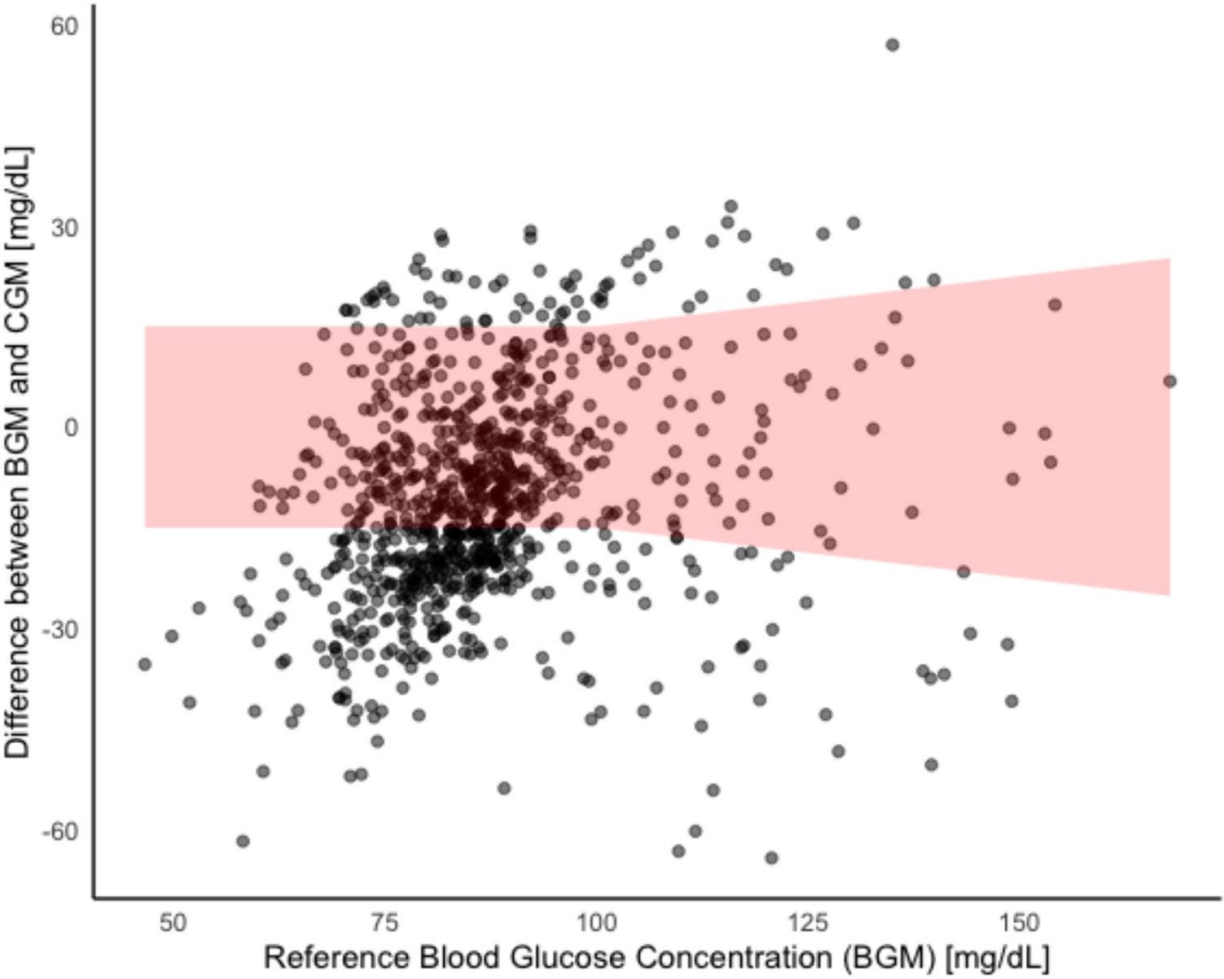
CGM accuracy for each individual BGM value showing the absolute difference between the CGM results and the BGM reference method. Red area indicates the ± 15 mg/dL for glucose concentrations < 100 mg/dL and the ± 15% for glucose concentrations ≥ 100 mg/dL per the ISO 15197:2013/EN ISO 15197:2015

Figure 4 highlights the results according to the analysis based on the standards by the ISO 15197:2013/EN ISO 15197:2015. Overall, 51.0% of the CGM measurements fall within the limits of ± 15.00 mg/dL for blood glucose concentrations < 100 mg/dL and 50.0% of the CGM measurements fall within the limits of ± 15.00% for blood glucose concentrations ≥ 100 mg/dL.

## Discussion

The current study aimed to investigate the accuracy of a CGM device compared to BGM for monitoring of blood glucose levels before, during and after an intense lower body strength training and the influence of a high-carbohydrate compared to a low-carbohydrate breakfast.

The primary finding of the current analysis indicates that while the Bland–Altman analysis demonstrated an acceptable level of agreement during lower body strength training (mean bias: − 8.96 mg/dL) and non-exercise phases (mean bias: − 7.95 to − 17.83 mg/dL), the agreement was lowest in the post-exercise phase (− 17.83 [− 20.75 to − 14.91] mg/dL). However, individual measurements can vary by up to ± 60 mg/dL.

**Fig. 5:**
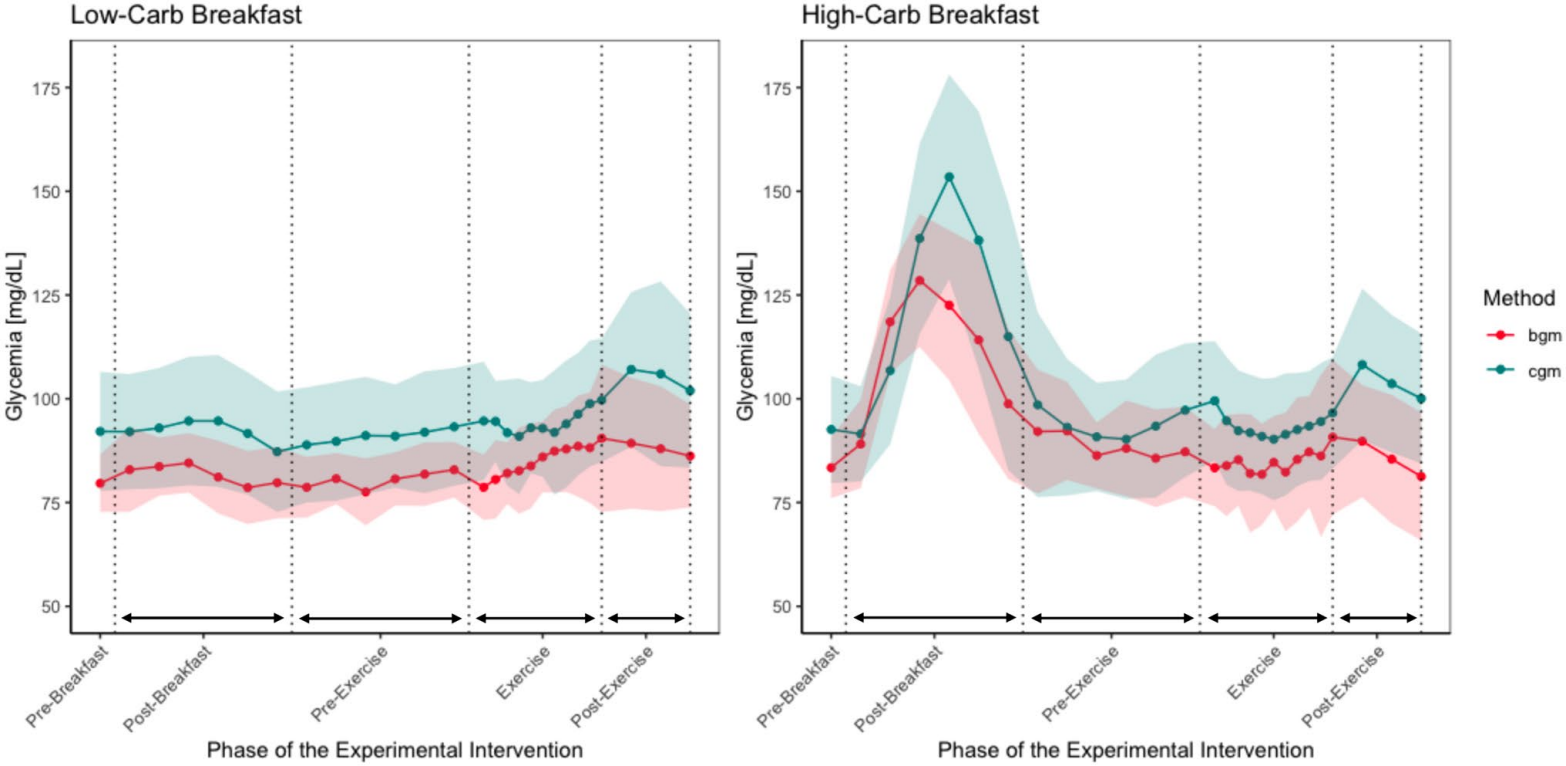
Mean and standard deviation of BGM and CGM measures over the course of the standardized interventions with respect to the different breakfast conditions

The results of the Bland–Altman analysis in our group of young adults without diabetes revealed no impact of the strength training regime on the accuracy of CGM. This is in contrast to findings of a comprehensive review in populations with diabetes (Munoz Fabra et al. 2021) as well as high-intensity interval running in recreational athletes without diabetes (Bauhaus et al. 2023; Clavel et al. 2022). In their study, Clavel et al. (2022) conducted the same nutritional intervention, augmented with high-intensity interval running training for the exercise component. They reported a moderate bias in the exercise phase, while biases in other phases were found to be trivial to small. Bauhaus and colleagues (Bauhaus et al. 2023) conducted a comparable intervention with six tests in total where two tests were conducted at rest in a fasted state, two tests conducted at rest following the intake of 1 g of glucose per kilogram of body weight, one test involving 60 min of moderate-intensity running after glucose intake, and one test involving high-intensity running following glucose intake. Here, exercise was initiated 30 min after dietary intake. The findings indicated that the CGM device had reduced validity during high-intensity training, while its accuracy improved during longer exercise durations and non-exercise periods, regardless of nutritional content. A previous study in individuals with type-1 diabetes showed that resistance exercise causes less initial decline in blood glucose during the activity, but is associated with more prolonged reductions in post-exercise glycemia than aerobic exercise (Yardley et al. 2013). The distinct difference in the glucose level behavior between resistance training and high-intensity endurance training may explain the differences in accuracy between ours and the findings of Clavel et al. (2022) and Bauhaus et al. (2023) (Fig. 5). As less accuracy of CGM devices has been reported especially during lower blood glucose levels (Bay et al. 2013; Diabetes Research in Children Network Study 2004), the accuracy of CGM systems during strength training may not be as significantly affected as it is during high-intensity endurance training. However, it is important to consider that these speculations are based on data from individuals with type-1 diabetes, who show markedly impaired glucose metabolism compared to individuals without diabetes. Furthermore, the shorter exercise phase duration in our study (approximately 25 min compared to 40–60 min) in the studies of Clavel et al. (2022) and Bauhaus et al. (2023) may explain the observed differences. Moreover, Clavel and co-workers implemented a finger prick self-monitoring device as a reference method which was from the same manufacturer (Abbott) as the tested CGM device, making an objective comparison of both measurement methods questionable. The explanations provided above also account for the observed discrepancy in the agreement between CGM and BGM during the post-exercise phase in our study, in contrast to the findings of Clavel et al. (2022), who reported only trivial to small biases in the post-exercise period following a high-intensity interval running intervention in individuals without diabetes. The study of Bauhaus et al. (2023) did not specifically analyze for the post-exercises’ period.

A visual analysis of the average CGM and BGM values throughout the experimental interventions suggests that CGM generally reflects and overestimates the rate and direction of BGM changes (Fig. 5). In this context, if an individual is unaware of the common overestimation by a continuous glucose monitoring device, they may fail to consume glucose or do so too late to prevent critically low blood glucose levels. Conversely, if the trend accuracy and overestimation are consistent and the individual is aware of this tendency, they may effectively mitigate potential hypoglycemic events. However, research on CGM accuracy is mixed. Some studies report that CGM devices underestimate blood glucose levels (Pleus et al. 2015; Price et al. 2023), while others indicate an overestimation (Bally et al. 2016; Clavel et al. 2022; Weinstein et al. 2007). These discrepancies may be due, among other factors, to the specific algorithms used by different CGM devices (Del Favero et al. 2014).

Furthermore, the examination of differences between CGM and BGM values for each data point across participants reveals significant interindividual variability in the accuracy of rate and direction of change, particularly during the exercise and post-exercise phases.

In previous analysis, the BGM demonstrated high accuracy, independent of concentration, with a precision of 3.5 ± 3.4% (Nowotny et al. 2012). However, the present comparisons between CGM and BGM measurements revealed fluctuations up to ± 60 mg/dL, particularly during the post-breakfast phase, as shown in Fig. 2B. This represents a deviation of approximately 62% from the average BGM value of 96.9 ± 22.2 mg/dL recorded during this period. Consequently, it can be inferred that the primary source of variation is attributable to the CGM measurements. Further sources of variance may be attributed to interindividual differences in skin temperature, shifts in interstitial fluid, and sweat production during exercise, among other factors (Laguna Sanz et al. 2019). Therefore, it is advisable to perform a comparative analysis of CGM responses and BGM measurements during training sessions before solely implementing CGM for blood glucose analysis during exercise.

In the present analysis, clinical accuracy was evaluated using three criteria: (i) EGA indicated that 50.5% to 64.3% of measurements fell within Zone A and 31.7% to 44.6% within Zone B, with increased failure to treat errors (hypoglycemia not detected) during and after exercise, (ii) MARD showed a percentage difference of 17.5 ± 13.2% to 24.3 ± 17.4%, and (iii) according to ISO 15197:2013/EN ISO 15197:2015 standards, only 51.0% of CGM measurements fell within ± 15.00 mg/dL for blood–glucose concentrations < 100 mg/dL, and 50.0% of CGM measurements fell within ± 15.0% for blood glucose concentrations ≥ 100 mg/dL.

These results fail to satisfy the established minimum criteria for glucose meters intended for use in populations with diabetes (Wernerman et al. 2014). These standards require that 99% of measurements fall within Zone A of the EGA (International Organization for Standardization, 2013), 95% of CGM measurements should fall within 15 mg/dl (for values below 100 mg/dl) or 15% (for values above 100 mg/dl) of the BGM reference for the standards of the ISO 15197:2013/EN ISO 15197:2015 (International Organization for Standardization 2013), and that MARD values are below 10% (Danne et al. 2017). Nevertheless, for individuals without diabetes engaged in strength training, such a high level of accuracy may not be critical. In this context, the primary concern is whether CGM measurements accurately reflect the rate and direction of blood glucose changes. This information enables exercisers to potentially counteract any decline in performance during prolonged resistance training sessions (Cholewa et al. 2019; King et al. 2022), by providing fast-digesting carbohydrates during training as a precaution before potentially reaching critically low blood glucose values.

To the best of the authors’ knowledge, the current investigation is the first to analyze the accuracy of a CGM device compared to BGM in healthy individuals during an intense lower body strength training preceded by meals containing different macronutrient intakes. Although the findings from this study offer important insights into the use of CGM during strength training and in connection with different pre-exercise macronutrient meals, it is critical to acknowledge that the training duration was limited to approximately 20 min and involved only ten sets of a single exercise. This special protocol does not reflect a typical strength training session for experienced exercisers, who usually engage in various exercises targeting different muscle groups and lasting between 45 and 90 min. The specific nature of this session, primarily aimed at inducing muscle hypertrophy, imposes distinct metabolic demands compared to other strength training methods (e.g., maximum strength, strength-endurance, plyometrics, etc.). Consequently, these results should not be directly generalized to other strength training regimes. Furthermore, although both sexes were included in this investigation, we did not conduct a statistical analysis specifically for sex-specific influences on CGM accuracy or individual variation, because (i) visual inspection of the data did not reveal any noticeable effects, and (ii) the sample size was too small to support such analysis. In general, it is important to note that to this date, there is no scientific evidence supporting the need of continuous measurement of blood glucose levels for healthy individuals without diabetes for optimizing strength training performance (Henselmans et al. 2022).

The amount of glucose present in the blood is quite limited, typically around 4–5 g in total. This level is tightly regulated within a narrow range, usually between 70 and 140 mg/dL, through a delicate balance of supply from the intestine and liver, and demand from cells, such as skeletal muscle. During exercise, the intensity and duration are the primary determinants in muscle glucose uptake, and glucose uptake from blood can be up to 100 times faster than they do at rest and account for up to 40% of oxidative metabolism during prolonged exercise, when muscle glycogen is depleted (Richter and Hargreaves 2013). It is important to understand that fluctuations in blood sugar levels are normal, such as experiencing hyperglycaemia during high-intensity exercise or post-exercise as a natural response, rather than indicative of impaired glucose regulation or a flawed fuelling strategy (Bowler et al. 2023). A recent study utilizing CGM has revealed interesting patterns among elite endurance athletes (Flockhart et al. 2021). These athletes spend more time below the normal glucose threshold of about 70 mg/dL (and above 50 mg/dL), especially in the early morning during sleep, and they also spend more time above the upper threshold of about 140 mg/dL, typically during the early afternoon, while they generally maintain blood glucose levels within the normal range during their training sessions. These data exemplify that although net blood glucose is easily measured with CGM and hypothetically can been used as a proxy for glucose availability and utilization in sport, circulating concentrations of glucose fail to illustrate the dynamic changes in rates of blood glucose appearance from the liver or gastrointestinal tract and subsequent uptake into tissues (Bowler et al. 2023). Thus, although there are numerous theoretical and anecdotal benefits associated with using CGM, there is currently no scientific consensus on how real-time blood sugar availability can effectively improve performance, given the lack of evidence supporting the effectiveness of CGMs in this context.

## Conclusion

At the group level, the findings from this study indicate that CGM provides acceptable accuracy before and during a brief, intense lower body strength training session in young individuals without diabetes who consumed either a high- or low-carbohydrate breakfast 2 h before exercising. However, accuracy declined in the post-exercise phase, with notable intraindividual variability observed. While the accuracy level may be adequate for healthy exercisers to assess the direction and magnitude of glucose fluctuations, it did not achieve the minimum clinical standards required for populations with diabetes. CGM could aid athletes without diabetes by tracking glucose fluctuations due to diet and exercise. Although utilization of CGM shows potential in gathering, analyzing, and interpreting interstitial glucose for improving performance, the application in sports nutrition is not yet validated, and challenges in data interpretation limit its adoption. Future research should explore CGM's utility in strength training for personalized nutritional decision-making (Hall et al. 2018) particularly during extended, glucose-depleting exercise sessions and/or periods of caloric deficit, especially in instances of actual hypoglycemia.

### Perspective

While CGM deployment may not be necessary for strength exercisers without diabetes and challenges in data accuracy and interpretation may hinder athletes' adoption of CGMs, the present findings highlight its utility in offering insights into blood glucose levels before and during strength training sessions. Practitioners can gain specific insights into how blood glucose reacts to various dietary intakes, across different workout durations and types, and during phases of caloric deficit, maintenance, or surplus. However, it is crucial for practitioners to recognize that the accuracy of CGM observed in this study does not reach the minimum clinical standards and exhibits significant interindividual variability, necessitating cautious application.

## Supplementary Information

Below is the link to the electronic supplementary material.

Supplementary file1 (XLSX 114 KB)

Supplementary file2 (XLSX 12 KB)

## Data Availability

All data generated or analysed during this study are included in this published article and its supplementary information files.

## Abbreviations

1RM: One-repetition maximum
BGM: Blood glucose measurement
CGM: Continuous glucose measurement
CI: Confidence intervals
EGA: Error grid analysis
High-Carb: High-carbohydrate
ISO: International organization for standardization
LoA: Limits of agreement
Low-Carb: Low-carbohydrate
MARD: Mean absolute relative difference
